# Galactose uncovers face recognition and mental images in congenital prosopagnosia: The first case report

**DOI:** 10.1179/1476830513Y.0000000091

**Published:** 2014-09

**Authors:** Janina Esins, Johannes Schultz, Isabelle Bülthoff, Ingo Kennerknecht

**Affiliations:** 1 Max Planck Institute for Biological Cybernetics, Tübingen, Germany; 2 Department of Psychology, Durham University, Durham, UK; 3 Department of Brain and Cognitive Engineering, Korea University, Seoul, Korea; 4 Institute of Human Genetics, Westfälische Wilhelms-Universität Münster, Münster, Germany

**Keywords:** Monosaccharide, Galactose, Congenital prosopagnosia, Developmental prosopagnosia, Face blindness, Face recognition, Mental imagery, ADHD

## Abstract

A woman in her early 40s with congenital prosopagnosia and attention deficit hyperactivity disorder observed for the first time sudden and extensive improvement of her face recognition abilities, mental imagery, and sense of navigation after galactose intake. This effect of galactose on prosopagnosia has never been reported before. Even if this effect is restricted to a subform of congenital prosopagnosia, galactose might improve the condition of other prosopagnosics. Congenital prosopagnosia, the inability to recognize other people by their face, has extensive negative impact on everyday life. It has a high prevalence of about 2.5%. Monosaccharides are known to have a positive impact on cognitive performance. Here, we report the case of a prosopagnosic woman for whom the daily intake of 5 g of galactose resulted in a remarkable improvement of her lifelong face blindness, along with improved sense of orientation and more vivid mental imagery. All these improvements vanished after discontinuing galactose intake. The self-reported effects of galactose were wide-ranging and remarkably strong but could not be reproduced for 16 other prosopagnosics tested. Indications about heterogeneity within prosopagnosia have been reported; this could explain the difficulty to find similar effects in other prosopagnosics. Detailed analyses of the effects of galactose in prosopagnosia might give more insight into the effects of galactose on human cognition in general. Galactose is cheap and easy to obtain, therefore, a systematic test of its positive effects on other cases of congenital prosopagnosia may be warranted.

Congenital prosopagnosia (CP) is the lifelong impairment in recognizing someone by their face. It is present from birth on, in contrast to acquired prosopagnosia which refers to the loss of an originally functioning face recognition system due to a brain lesion. CP is very common with a prevalence of 2.5% in the general population^1^ and thus represents the majority of prosopagnosia cases.^2^ The general processes or impairments causing CP are not yet understood, but accumulating evidence suggests that CP is hereditary because it almost always runs in families.^1,3,4^

## Report of the case

In March 2008, a 40-year-old woman, LI, contacted us because she was unable and never has been able to recognize the faces of friends or even of her close family members. The self-reported diagnosis of CP (also known as face blindness) in the absence of any known events of brain damage or malformation was supported by us with a semi-structured diagnostic interview as described elsewhere.^5^ In addition, LI had previously been diagnosed with attention deficit hyperactivity disorder (ADHD) by a medical unit elsewhere.

**Figure 1:**
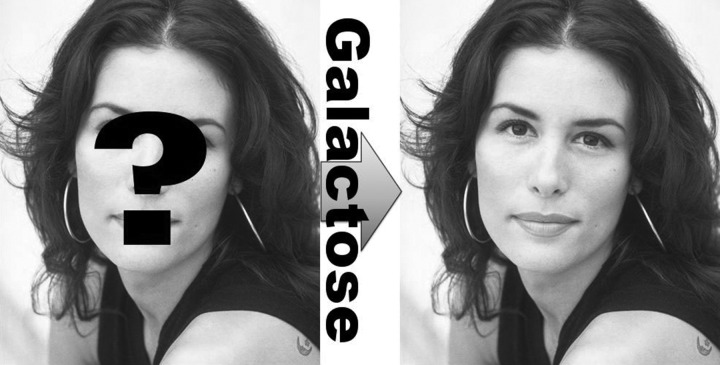
Artist's impression of the impact of galactose on face blindness.

In June 2011, LI had a very curious experience and contacted us a second time. On advice from a friend, she started the daily intake of one tablespoon (about 5 g) of d-galactose as self-medication for her ADHD. Three days after the start of galactose intake, she began noticing clear changes in her ADHD-related symptoms. However, most surprisingly, her prosopagnosia-related symptoms improved as well; LI could suddenly recognize the faces of her family members and of persons that she had encountered repeatedly but had always failed to recognize. She could even recognize the faces of people she had not seen for many years. Furthermore, she experienced mental images for the first time in her life. LI was even somehow afraid of the vivid and colorful dreams she now had, as this was a new experience for her. Additionally, she reported an improved sense of orientation, and her need for sleep was normalized from at most 4 hours to about 8 hours per night.

A third meeting took place 3 months later, 4 weeks after she stopped her daily galactose intake following our request. At this meeting, she reported that all the effects of galactose intake had disappeared, and now described the loss of her newly acquired face recognition abilities and mental imagery, a significantly reduced need for sleep, and a decreased sense of orientation.

To assess the generality of our observation, we asked 16 further congenital prosopagnosics to take a daily dose of 5 g of galactose for 7 days and report any changes. Three of those participants had also been diagnosed with ADHD. None of the 16 participants observed any noticeable effects of galactose intake.

## Comment

Our participant LI showed the habitual, previously reported symptoms of CP: impaired face recognition^6^, weak sense of navigation^6^, and a lack of mental images, at least of faces.^7^ The intake of monosaccharides is related to improvements in cognitive performance, but descriptions of their influence on visual perception especially have not been published so far.^8^ Other studies have shown other positive effects of galactose on brain function. A study by Best *et al.*^9^ reported a positive effect of the supplementation of saccharides including galactose and other monosaccharides on memory performance and well-being. Further work has revealed that galactose is important for the healthy functioning of the human brain, and animal studies have shown that galactose is essential for proper myelin formation, which is necessary for functional insulation of the axons of nerve cells.^10^

Galactose could play an intricate role in visual cognition. For LI, daily oral intake of 5 g of this monosaccharide had several very positive effects on her life. A possible explanation for our findings is that LI suffers from a (relative) deficiency in galactose that impairs face recognition and the generation of mental images. Supplementing galactose might compensate, a relative receptor insufficiency and alleviate her symptoms. Further, the fact that LI was able to recognize previously unrecognizable faces after taking galactose implies that familiar faces were stored in her memory but remained inaccessible before galactose supplementation (Fig. 1).

A very interesting aspect of our case is that the described effects had not occurred prior to galactose intake. Glucose, another monosaccharide and part of LI's normal diet, did not show any effects. Ritalin, which LI took to improve her ADHD symptoms before she switched to galactose, did not have the same impact either. The fact that none of the 16 other tested prosopagnosics showed similar responses to galactose as LI is in line with strong evidence that prosopagnosia is phenotypically and genetically heterogeneous,^5^ which might explain why only (very) few prosopagnosics seem to benefit from galactose.

This as yet unreported effect of galactose on prosopagnosia raises many hopes. If galactose proves to be helpful for (albeit rare) subtypes of CP, it would provide a simple and cheap way to immensely improve the life of the people concerned.

## Ethics

The study was approved by the ethical committee of the University of Münster, Germany, protocol No 3XKenn2, amendment 2012-512-f–S. Informed consent from all participants was obtained.
